# Odontological Society of Pennsylvania

**Published:** 1895-11

**Authors:** 

**Affiliations:** Odontological Society


### ODONTOLOGICAL SOCIETY OF PENNSYLVANIA.

The regular meeting of this Society was held September 14, 1895. At the close of routine business the paper of the evening was read by Dr. C. N. Peirce, entitled "What has been done by the Profession in France on Pyorrhœa Alveolaris?"

(For Dr. Peirce's paper, see page 660.)

Dr. Brubaker was called upon by the President, who responded as follows:

It will have been noticed in the abstract of Professor Magitot's paper, as read by Professor Peirce, that pyorrhœa alveolaris has been observed in the diabetic, the albuminuric, as well as in the arthrotic or gouty patients. The occurrence of pyorrhœa in diseases which are apparently so widely separated as these three would seem to militate against the view that the disease in question was simply a gouty manifestation. If, however, the gouty diathesis be received in its totality, it will be observed that very frequently both glycosuria and albuminuria are but two more of its various manifestations.

In many cases of incomplete gout the excretion of sugar by the kidneys alternates with deposits of uric acid, and as the production of sugar is supposed to be a function of the liver, the inference is that this disturbance of function is dependent upon uratic deposits in the liver. With the elimination of large quantities of water under such circumstances the uratic salts are excreted, after which the gouty symptoms subside. If a patient with this temporary glycosuria had at the same time pyorrhœa, it might, at first glance, be supposed that the former was the cause of the latter, rather than that they were both symptoms of one systemic condition.

The same holds true with reference to the transient form of albuminuria. Traces of albumen are exceedingly common in the gouty condition, and are supposed to be due to the involvement of the kidney, and more particularly the excreting epithelium from the excess of uric acid. Here, again, the two conditions, albuminuria and pyorrhœa, do not necessarily stand in the relation of cause and effect, but as simultaneous expressions of a common diathesis.

*Dr. Head.*—The treatment by a silk seton, as suggested by De Serran, seems most valuable, and should be given a fair trial. The difficulty of healing the pockets is increased by the presence of pus at the bottom, and if the pus should be allowed to escape it is reasonable to suppose that the granulation would be much more effective and rapid.

*Dr. Peirce.*—I was in hopes that Dr. York would be here this evening. He has a very interesting case that has been under his care, which I have seen three or four times,—a lady whose teeth were very loose and the gums very much absorbed. Some four or five months ago, while consulting with him, I suggested to her that she use considerable friction with her finger on the gums every day, rubbing them freely around where the parts had been so much destroyed. At her last visit she assured me she had done this vigorously, and the change was remarkable. I have constantly taken the ground that we cannot produce tissue that has been once absorbed, but the gum tissue in that patient's mouth was normal, probably induced by the friction of her finger. Certainly the remedies applied would not have acted so, but the friction which she gave them undoubtedly brought about the development of a normal quantity of good, firm tissue.

JOSEPH HEAD, M.D., D.D.S.,
*Editor Odontological Society.*

